# Genomic prediction of beef quality using GWAS-prioritized markers

**DOI:** 10.1093/tas/txaf175

**Published:** 2026-01-06

**Authors:** Gabriel A Zayas, Raluca G Mateescu

**Affiliations:** Department of Animal Sciences, University of Florida, Gainesville, Florida, United States; Department of Animal Sciences, University of Florida, Gainesville, Florida, United States

**Keywords:** Brangus, *CAPN1*, genomic prediction, GWAS, marbling, tenderness

## Abstract

Tenderness and marbling are key carcass quality traits in beef cattle that strongly influence consumer eating satisfaction and repeat purchasing behavior. Because both traits are measured postmortem, they are difficult to incorporate into routine selection programs. Genomic selection therefore provides a practical strategy to improve these traits. This study evaluated the effectiveness of GWAS-informed SNP preselection for predicting Warner–Bratzler shear force (WBSF) and marbling breeding values using reduced marker densities in Brangus cattle. Using a structured population (*N* = 1066), we conducted a 10-fold cross-validation with SNP subsets ranked by GWAS significance and compared them to 10 random SNP subsets of the same number and full SNP panel. External validation was performed using 338 animals from an independent source. For WBSF, small panels (eg top 50 SNPs) achieved accuracy comparable to the full panel, driven by a strong QTL on chromosome 29 (*CAPN1*). In contrast, marbling required broader marker coverage for optimal prediction, consistent with a polygenic trait architecture. Across all subset sizes, GWAS-ranked SNPs outperformed random subsets, and in several cases matched full panel accuracy. External validation confirmed the reliability of these results. These results demonstrate that trait-specific genetic architecture strongly influences the marker density required for reliable genomic prediction and highlight the value of GWAS-informed SNP prioritization for optimizing genomic prediction strategies in crossbred cattle.

## Teaser

Reduced GWAS-informed SNP sets deliver competitive prediction for tenderness and highlight trait-specific differences in marker count requirements.

## Introduction

Meat quality traits such as marbling and tenderness are key determinants of eating quality, influencing flavor, juiciness, and overall palatability, which in turn affect consumer satisfaction and repeat purchasing behavior (Mateescu et al. 2015; Tatum 2015). Despite their economic and biological importance, both marbling and tenderness remain difficult to evaluate because they are typically measured postmortem. Marbling can be indirectly assessed in vivo using ultrasound-based estimates of intramuscular fat, but no equivalent practical proxy exists for tenderness. The most widely accepted measure of tenderness, Warner-Bratzler shear force (WBSF), objectively quantifies the force required to shear a standardized, cooked muscle core, and serves as an established indicator of consumer-perceived tenderness. However, because WBSF requires physical carcass samples, its application is limited in live-animal selection (Golden 2021).

Because consumers consistently rank tenderness among the most important factors in beef eating satisfaction, genetic improvement of this trait could enhance product consistency, reduce dissatisfaction, and support higher value premiums across markets (NCBA 2020a). Marbling, in turn, contributes to both flavor and visual appeal and is widely used in branded beef programs to signal quality to consumers (NCBA 2020b). Consequently, both traits represent critical targets for genetic improvement. Previous studies have reported moderate to high heritability for both traits, with marbling estimates ranging from 0.30 to 0.67 and WBSF from 0.19 to 0.45, depending on population and methodology (MacNeil et al. 2010; McClure et al. 2012; Mateescu et al. 2015; Zayas et al. 2024). These estimates indicate substantial potential for genetic improvement if effective selection tools can be implemented.

To overcome the limitations of traditional phenotyping, genomic prediction has emerged as a promising strategy for improving difficult-to-measure traits (Wu et al. 2016; VanRaden et al. 2020). In this study, the term *genomic estimated breeding value* (GEBV) refers to genomic predictions obtained from SNP-based mixed models that estimate animal genetic merit using genome-wide marker information. These GEBVs are derived exclusively from genotype and phenotype data using a GBLUP or SNP-BLUP framework and do not incorporate pedigree information. Accordingly, they should not be interpreted as genomically enhanced EPDs (GE-EPD) as defined by industry evaluation systems, nor as molecular breeding values (MBV) based on a small number of fixed markers.

Early marker-assisted selection (MAS) efforts, such as the STAR system developed to rank cattle based on tenderness-associated SNPs, demonstrated the potential of DNA information in selection but were constrained by their reliance on a small number of markers with modest predictive ability (Johnston and Graser 2010). These early MAS panels for beef tenderness focused on polymorphisms in genes involved in postmortem proteolysis, particularly calpain-1 (*CAPN1*) and its inhibitor calpastatin (*CAST*). Several studies demonstrated consistent associations between *CAPN1* and *CAST* variants and Warner–Bratzler shear force across *Bos taurus, Bos indicus*, and crossbred cattle (Page et al. 2002; White et al. 2005; Schenkel et al. 2006; McClure et al. 2012). While these markers were successfully incorporated into early commercial tests, their predictive ability was limited by varying allele frequencies, incomplete LD with causal variants and population-specific effects (Corva et al. 2007; Curi et al. 2010; Tait et al. 2014; Sun et al. 2018). Subsequent genomic selection approaches replaced fixed-effect markers with genome-wide random effects, improving robustness but increasing genotyping density requirements. However, the cost of high-density panels and their limited transferability across diverse breeds may constrain their widespread use in commercial cattle populations (Kelly and John 2014; Weber 2020).

Moreover, most prediction models are trained on *Bos taurus taurus* reference populations such as Angus or Hereford and tested under relatively uniform conditions. Brangus cattle—a composite breed derived from Brahman and Angus—are used across both seedstock and commercial herds spanning diverse, often subtropical environments, where G × E effects can be pronounced.

The Brangus breed’s distinctive linkage disequilibrium patterns and allele frequencies differ substantially from purebred *Bos taurus* breeds, which can reduce the accuracy of genomic predictions trained on conventional datasets (Zayas and Mateescu 2025). As a result, prediction accuracy often declines when models developed in purebred populations are applied to crossbred animals like Brangus due to genetic and environmental mismatches (Olson et al. 2012; Kachman et al. 2013). In addition, large panels may include SNPs with weak or population-specific effects that introduce noise rather than improve predictive power (van Binsbergen et al. 2015; Wu et al. 2016).

One strategy to address these challenges is to reduce SNP panel size by preselecting markers with strong genome-wide association study (GWAS) signals for a given trait. This approach has the potential to retain much of the predictive power of high-density panels while reducing genotyping costs and improving model portability across diverse populations (Wu et al. 2016). Conceptually similar to MAS, GWAS-informed marker selection may improve prediction robustness in commercial settings by focusing on informative loci while minimizing noise and avoiding overfitting to training-specific artifacts. Although using fewer markers may modestly reduce theoretical accuracy, this tradeoff may be offset by increased stability of predictions, and potentially broader applicability across populations.

Despite its promise, relatively few studies have systematically evaluated how reducing SNP density via ranked SNP subsets impacts GEBV, particularly for meat quality traits in composite breeds.

This study aimed to evaluate the impact of GWAS-informed SNP preselection on the accuracy of GEBVs for marbling and WBSF in Brangus cattle. Specifically, we examined the relationship between marker density and predictive performance for these two economically important meat quality traits. Prediction performance (accuracy and bias) was assessed using two complementary approaches: (1) a cross-validation design within a population with well-defined contemporary groups, and (2) external validation in an independent population lacking estimable contemporary groups, to mimic commercial or producer-level scenarios where environmental structure is unknown or inconsistent. This study’s goal is to determine the minimum number of informative SNPs required to maintain robust prediction. The emphasis is not on maximizing absolute prediction accuracy, but rather on evaluating whether substantially reduced marker sets, anchored by statistically supported loci, can deliver accuracy comparable to full-density panels.

## Material and methods

### Animals and phenotypic measurements

All experimental procedures involving University of Florida–managed animals (UF Multibreed Angus–Brahman population) were approved by the University of Florida Institutional Animal Care and Use Committee (protocol #201003744). Seminole Tribe cattle were managed and harvested under standard commercial production practices at a USDA-inspected facility, and no experimental animal procedures were performed on these animals. The primary study population consisted of 1066 Brangus steers born in 2014 and 2015 and raised by the Seminole Tribe of Florida, Inc. Cattle were finished at a commercial feedlot (Quincey Cattle Company, Chiefland, FL) on a corn-based diet formulated with supplemental protein, vitamins, and minerals. Steers were harvested under USDA FSIS inspection at a commercial packing plant at approximately 2 yr of age. (FPL Food LLC, Augusta, GA) (Humane Methods of Livestock Slaughter, 7 U.S.C. Chapter 48, 1958).

Production characteristics and additional details regarding sample collection and processing were reported previously (Rodriguez et al. 2023). Briefly, carcasses were ribbed between the 12th and 13th ribs at 48 hours postmortem. Marbling score (MARB) was evaluated by trained personnel following USDA grading guidelines (Hale et al. 2013), and Warner-Bratzler Shear Force (WBSF) was measured on cores collected from cooked longissimus dorsi steaks after 14 days of aging.

Contemporary groups (CG) were formed based on feedlot pen nested within ranch origin (23 ranches). For a more in-depth description of animals and CG please see (Rodriguez et al. 2023). To ensure reliable estimates, only CGs with at least five animals were retained, resulting in 968 steers assigned to 32 CGs. Among these, 950 animals had MARB records and 940 had WBSF records.

An external validation population (*n* = 338) was used to evaluate prediction performance in an independent context. This population consisted of animals from the University of Florida Multibreed Angus-Brahman (UF MAB) herd, a resource population developed to capture a broad spectrum of Brahman–Angus breed composition. Animals were harvested between 2015 and 2019 and phenotyped under standardized protocols to ensure consistent evaluation of carcass traits (Leal-Gutiérrez et al. 2018b). Within this external dataset, 321 animals had MARB records and 319 had WBSF measurements available. Due to the diversity of origins and sparse group sizes, CGs were not assigned however we did control for year.

### Genotyping and quality control

DNA was extracted from blood or tissue samples using the QIAamp DNA Mini Kit (Qiagen, Valencia, CA, USA) according to the manufacturer’s protocol and stored at −20°C. Genotyping was performed using the Bovine GGP F250 SNP array (Neogen GeneSeek, Lincoln, NE, USA), which includes 221,115 markers enriched for functional variants such as non-synonymous, frameshift, and stop codon mutations. Only autosomal SNPs mapped to the ARS-UCD1.2 bovine genome assembly were retained for analysis.

Quality control (QC) filtering was conducted using PLINK v2.00a5LM (Chang et al. 2015). For both the MARB and WBSF datasets, samples were excluded if they had a genotype call rate below 90%, and SNPs were removed if they had a call rate below 90% or a minor allele frequency less than 5%. After filtering, the MARB dataset retained 939 animals and 111,085 SNPs, while the WBSF dataset included 930 animals and 111,142 SNPs.

### Estimation of genetic parameters

GEBVs for MARB and WBSF were predicted using the genomic best linear unbiased prediction (GBLUP) approach implemented in the BLUPF90 software suite (Misztal et al. 2014).The model included fixed effects for CG, and a random additive genetic effect modeled using a genomic relationship matrix (**G**) derived from post-QC SNP genotypes.

The mixed model took the following form:


y=Xb + Zu+e

Where:

-
y is a vector of phenotypes;
-
X  and  Z  are incidence matrices relating records to fixed effects (b) and random animal genetic effects (u), respectively;
- *b* is a vector of the fixed effects which included the previously mentioned CG (n = 32).
-
u indicates a vector of random animal additive effects. These effects are distributed as $u\;∼\;N(0,G\sigma_{u}^{2})$, where $\sigma_{u}^{2}$ is the additive genetic variance. Where G is the genomic relationship matrix G was constructed based on the method proposed by VanRaden (VanRaden 2008).
- *e* is a random residual vector, distributed $e\;∼\;N(0,I\sigma_{e}^{2})$, with $\sigma_{e}^{2}$ signifying residual variance and **I** the identity matrix.

This model was used for the cross-validation scheme below to estimate variance components using *airemlf90* and implement *postGSf90* to estimate SNP effects (Misztal et al. 2014). The resulting predicted additive genetic effects from this SNP-based GBLUP framework are referred to throughout the manuscript as GEBVs.

### Cross-validation and external validation framework

To evaluate genomic prediction performance and assess the impact of SNP preselection on model accuracy, a genomic prediction pipeline was implemented consisting of cross-validation (CV) within the Brangus population and external validation in an independent population (UF MAB).

A 10-fold cross-validation framework was implemented using Brangus animals with valid CG assignments (Fig. 1.1–1.2). The Brangus population (hereafter referred to as CV population) was randomly partitioned into 10 folds, stratified by CG to ensure balanced representation of management and environmental effects. For each replicate, GBLUP models were trained on 90% of the data (nine folds) and used to predict GEBVs for the remaining 10% (test fold; Fig. 1.2). This procedure was repeated across 10 independent cross-validation replicates, each based on a different random fold assignment.

**Fig. 1.**
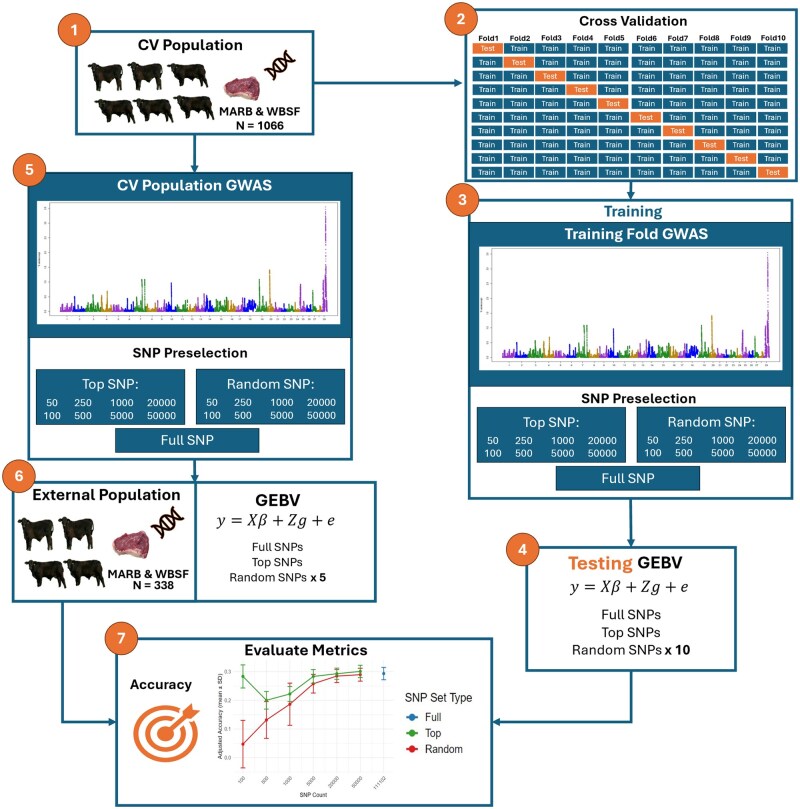
Workflow for evaluating the impact of GWAS-informed SNP selection on genomic prediction of carcass traits in Brangus cattle. (1) A structured cross-validation (CV) population (*N* = 1066) with phenotype and genotype data was used. (2) Cross-validation was performed by stratifying individuals into folds based on contemporary group. (3) Genome-wide association study (GWAS) was conducted within each training fold to rank SNPs based on association with the trait. (4) Genomic estimated breeding values (GEBVs) were computed for each test fold using the full SNP set, top-ranked SNP subsets (eg top 50–50,000), and randomly selected SNP subsets matched in size. (5) For the external population evaluation we used the full CV population to preselect SNP. (6) The external validation population (*N* = 338) was used to assess model portability across unstructured or independent groups. (7) Accuracy and bias metrics were evaluated across all SNP selection strategies and subset sizes to assess predictive performance.

**Fig. 2.**
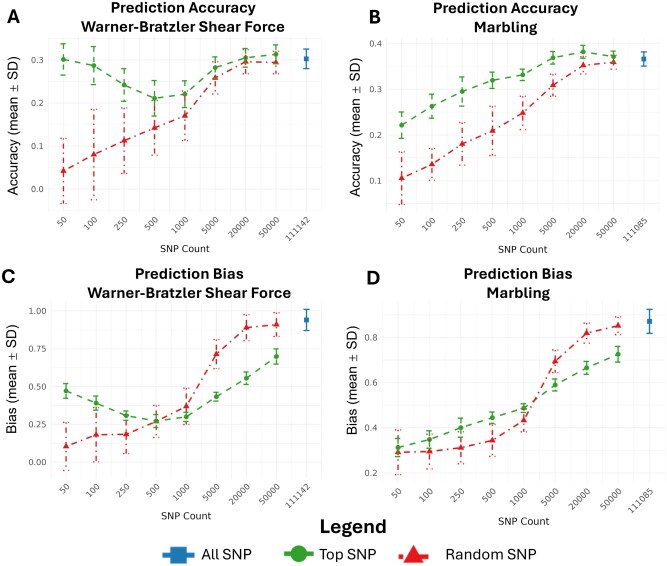
Accuracy and bias of genomic predictions across SNP subsets. Prediction accuracy (panel A: WBSF, panel B: MARB) and regression slope (panel C: WBSF, panel D: MARB) across SNP subset sizes. Blue dots represent the full SNP panel, while lines show the mean ± SE across CV replicates for top-ranked SNPs (green) and random SNPs (red). Smaller SNP subsets yielded comparable prediction accuracy to the full model for WBSF, whereas MARB showed improved accuracy with increasing SNP count. Regression slopes closer to 1 indicate lower bias; full SNP models showed the least bias across traits.

Within each training fold, variance components and heritability were first estimated using *airemlf90* (Misztal et al. 2014). GWAS were then conducted using SNP effect estimates derived from GBLUP using *blupf90* and *postGSf90* (Misztal et al. 2014). SNP solutions were extracted from the *postGSf90* output (Misztal et al. 2014; Aguilar et al. 2019) and markers were ranked based on –log_10_(*P*) values. Based on these rankings, fixed-size SNP subsets were created, including the top 50, 100, 250, 500, 1000, 5000, 20,000 and 50,000 markers. To provide a benchmarking baseline, random SNP subsets of equivalent sizes were also generated (10 replicates each). GEBVs were then estimated using *predf90*, which applies the fitted SNP solutions estimated from *postGSf90* to generate predictions for test animals (Misztal et al. 2014).

To assess the transferability of genomic prediction models, GEBV accuracy was evaluated in an independent external validation population (*n* = 338, UF MAB) using the entire cross-validation population as the training set. The external validation animals were not included in model training or GWAS analyses.

The same modeling and SNP preselection workflow described above were applied, with variance components, SNP effects, and SNP rankings estimated using the full Brangus population. GEBVs for UF MAB animals were then generated using *predf90* for the full SNP panel, top-ranked SNP subsets, and randomly sampled SNP subsets of matching sizes (Misztal et al. 2014).

### Evaluation of prediction metrics

Prediction accuracy was assessed using the formula:


$$\mathrm{Accuracy}=\frac{\mathrm{cor}(\mathrm{GEBV},\hat{y})}{\sqrt{h^{2}}}$$

(Pryce et al. 2012)

Prediction accuracy was estimated by comparing GEBVs to appropriately adjusted phenotypes ($\hat{y}$) in the validation population (Pryce et al. 2012; Warburton et al. 2023). In the cross-validation framework, phenotypes were adjusted for the fixed effects included in the GBLUP model, specifically CG, by fitting a linear model and using the resulting residuals as adjusted phenotypes. The Spearman correlation between the CG-adjusted phenotypes and the corresponding GEBVs was calculated within each fold and averaged across the 10 replicates to quantify predictive accuracy.

For the external validation, phenotypes were adjusted for harvest year to account for environmental and management effects not included in the training model. The resulting residuals were used to compute the correlation between observed phenotype and predicted GEBV in the external dataset.

To evaluate prediction bias, we estimated the regression slope of adjusted phenotype on GEBV. A slope equal to 1 indicates unbiased predictions. Slopes below 1 indicate overprediction of genetic differences, meaning that GEBVs are more widely dispersed than the observed phenotypes. Slopes above 1 indicate underprediction, meaning that GEBVs underestimate the true phenotypic differences. Bias was assessed both within cross-validation folds and in the external dataset. All accuracy and bias metrics were summarized across cross-validation replicates and SNP selection strategies and were visualized to evaluate trends in prediction performance across marker densities and population contexts.

### Marker-assisted prediction for WBSF

Given the consistent prediction accuracy observed for WBSF at very low SNP densities in both cross-validation and external validation, we further evaluated whether a targeted marker-assisted prediction approach could capture a substantial proportion of the predictive signal for this trait.

For each cross-validation replicate and fold, the single most significant SNP (lowest *P*-value) for WBSF was selected from the training population GWAS. A marker-assisted prediction model was then fitted using a linear model in R (R Core Team 2023), which included the selected SNP as a fixed effect, alongside the same fixed effects used in the GBLUP model (CG). The model took the form:


y=Xb + g×SNP+e

where y is the phenotype vector, X is the incidence matrix for fixed effects, b is the vector of fixed-effect coefficients, SNP represents the genotype dosage (0, 1, or 2) of the selected marker, g is the estimated allelic substitution effect, and e is the residual error term.

Prediction accuracy within the cross-validation population was evaluated by estimating SNP effects in the training set and predicting phenotypes in the held-out fold. Accuracy and bias were assessed using the same adjusted phenotypes and evaluation metrics described above.

To assess external performance, all unique SNPs identified across replicates and folds were compiled. SNP effects were then re-estimated using the entire cross-validation population, and marker-assisted predictions were generated for animals in the external validation population using these fixed-effect SNP models. Prediction accuracy and bias were evaluated using adjusted phenotypes in the external population, consistent with the genomic prediction analyses.

All MAS analyses were conducted in R, and results were summarized and visualized using ggplot2 (Wickham 2009).

## Results

### Trait distributions and heritability in the structured population

Descriptive statistics for WBSF and MARB are shown in Table 1. In the cross-validation training population, MARB ranged from 210 to 850 with a mean of 438.7 ± 84.6, while WBSF values ranged from 2.75 to 10.04 kg, averaging 5.05 ± 0.95. The external validation cohort showed similar distributions, though with slightly narrower ranges: WBSF values ranged from 2.1 to 8.0 kg (mean 4.83 ± 1.22), and MARB scores from 280 to 660 (mean 411.0 ± 83.1), indicating overall consistency in phenotype structure across populations. Genetic parameter estimates obtained from univariate GBLUP models are reported in Table 2, with fold-specific heritability shown in Fig. S1. MARB exhibited a higher genomic heritability (h^2^ = 0.52 ± 0.086) than WBSF (h^2^ = 0.34 ± 0.090).

**Table 1.** Descriptive statistics for marbling (MARB) and Warner–Bratzler shear force (WBSF) in training and external validation populations.

Population	Trait	N	Mean	SD	Min	Max	
**Training**	MARB	939	438.74	84.65	210.00	850.00	
**External**	MARB	321	411.00	83.08	280.00	660.00	
**Training**	WBSF (kg)	930	5.05	0.95	2.75	10.04	
**External**	WBSF (kg)	319	4.83	1.22	2.10	8.01	

Data includes the number of animals (*N*), mean values, standard deviation (SD), minimum and maximum values. Units are provided for each trait where applicable.

**Table 2.** Variance components and heritability estimates (± SE) for marbling (MARB) and Warner–Bratzler shear force (WBSF) in the training population.

Trait	V_G_	V_e_	V_P_	h^2^ (se)	
**MARB**	3435.6 ± 647.4	3166.0 ± 518.6	6601.60	0.520 ± 0.086	
**WBSF**	0.296 ± 0.083	0.573 ± 0.073	0.87	0.340 ± 0.090	

V_G_ is the additive genetic variance, V_e_ is the residual variance, V_P_ is the phenotypic variance, and h² is the heritability estimate.

### Influence of SNP subset size on GEBV accuracy in cross-validation

Figure 2 summarizes the prediction accuracy (top panels) and regression bias (bottom panels) across a range of SNP subset sizes for WBSF (left) and MARB (right). The full SNP models using all available markers (111,142 for WBSF and 111,085 for MARB) are shown as blue markers and serve as reference benchmarks.

For WBSF (Fig. 2A), the model using the full SNP panel achieved an average prediction accuracy of 0.303 ± 0.023. Notably, the top 50 SNPs achieved nearly identical accuracy (0.301 ± 0.036), suggesting that a small number of highly informative markers captured most of the predictive signal. Accuracy declined at intermediate subset sizes (100–1000 SNPs), before recovering at 5000 SNPs (0.282 ± 0.025), approaching the accuracy of both the top 50 and full SNP sets. Across all subsets below 20,000, top-ranked SNPs consistently outperformed randomly sampled SNPs of the same size, underscoring the value of GWAS-informed marker prioritization. The accuracies for the random subset and top subset begin to converge under large SNP subsets performing similarly to accuracy using the entire SNP Panel.

In contrast, MARB displayed a more gradual increase in accuracy with SNP count (Fig. 2B). The lowest accuracy was observed for the 50 SNP subset, while accuracy improved steadily with additional markers. The top 20,000 SNPs yielded the highest accuracy (0.382 ± 0.014), closely matching the full SNP set (0.366 ± 0.016).

Bias estimates (Fig. 2C–D) revealed overestimation of GEBV (slope <1) across most SNP subsets. At low SNP counts, top-ranked SNP subsets consistently exhibited less overestimation than random subsets. This likely reflects the fact that GWAS-prioritized panels capture markers with strong effects, which improves differentiation when marker density is very small. In contrast, randomly sampled subsets lack targeted trait-associated variants at these sizes and show greater overestimation of genetic differences or inability to estimate genetic differences. As SNP counts increased, this pattern reversed. For both WBSF and MARB, random subsets of above 1000 SNPs began to show less overestimation than their selected counterparts. These cross-validation bias patterns point to two complementary mechanisms. First, top-ranked SNPs provide better estimation at low densities because they include the strongest trait-associated loci. Second, as SNP count increases, random subsets increasingly capture broader polygenic signal, which reduces overestimation of GEBV differences and improves bias estimates.

### SNP prioritization based on GWAS signal

**Fig. 3.**
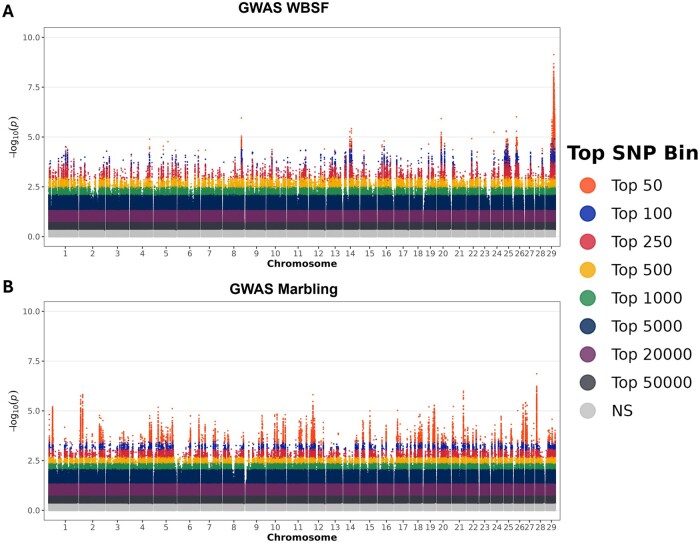
SNP Inclusion frequency and *P*-value stability across cross-validation replicates. Manhattan plots showing SNP association strength (–log_10_(*P*)) and bin inclusion across top-ranked SNP subset sizes in the genomic prediction pipeline. SNPs are colored based on their highest inclusion bin across cross-validation runs, from the top 50 (orange) to the top 50,000 (dark gray), with non-selected SNPs shown in light gray (NS). Panel A displays results for Warner–Bratzler shear force (WBSF) and panel B for marbling (MARB). Peaks represent regions with consistently strong associations and repeated inclusion across replicates.

To further investigate the genomic basis of prediction accuracy, the consistency and strength of SNP associations across cross-validation replicates were examined. Figure 3 shows the –log_10_(*P*) values for all markers across all folds and replicates, where each SNP is represented 100 times across 100 GWAS runs (10 folds x 10 replicates). The SNP is color-coded by its highest rank within a specific GWAS, allowing visualization of the genomic distribution of predictive signal and the degree of the redundancy or dispersion among top-ranked markers.

Table 3 summarizes the top 10 SNPs ranked by frequency of inclusion in the top 50 across replicates, along with their median *P*-values. For WBSF (Fig. 3A), a dense cluster of highly significant SNPs was observed on chromosome 29, particularly within the 42.3–43.4 Mb region. Many of these markers had near-identical *P*-values and appeared in the top 50 subset in 100% of cross-validation replicates. To avoid redundancy in downstream reporting, only one representative SNP from this region (rs110009059) was retained in the final top 10 SNP list (Table 3). This marker had a median –log_10_(*P*) of 6.95, with a range from 5.72 to 8.92 across replicates—the strongest signal of all SNPs evaluated. Additional highly ranked WBSF markers were identified on chromosomes 8, 14, 20, 22, 25, and 26, although with slightly lower signal intensity. Importantly, top-ranked SNPs showed high stability across replicates, with most appearing in ≥90% of the top 100 or top 250 bins.

**Table 3.** Top 10 SNPs ranked by inclusion on top ranked SNPs and median log10(*P*) across cross validation runs for WBSF and MARB.

Trait	SNP	BTA	BP	Median	SE	Min	Max	Top	Top	Top	Top	
				−log10(*P*)	−log10(*P*)	−log10(*P*)	−log10(*P*)	50 (%)	100 (%)	250 (%)	500 (%)	
**WBSF**	rs110009059	29	42914207	6.95	0.07	5.72	8.92	100%	100%	100%	100%	
**WBSF**	rs109460324	20	21223790	4.23	0.05	3.09	5.92	40%	81%	100%	100%	
**WBSF**	rs133572159	26	12866008	4.03	0.05	2.77	5.36	22%	75%	99%	100%	
**WBSF**	rs207746333	14	41546971	3.99	0.06	2.80	5.24	24%	68%	99%	100%	
**WBSF**	rs111015081	8	98789305	3.91	0.06	2.84	5.34	21%	66%	98%	100%	
**WBSF**	rs109110916	14	32693406	3.85	0.06	2.39	5.30	22%	55%	95%	99%	
**WBSF**	rs110541595	25	16436019	3.83	0.05	2.90	5.30	12%	57%	98%	100%	
**WBSF**	rs108984700	26	16642091	3.78	0.05	2.55	5.01	11%	53%	93%	100%	
**WBSF**	rs472434135	25	9441665	3.74	0.06	2.24	5.78	18%	47%	92%	98%	
**WBSF**	rs137699279	22	25982679	3.39	0.05	2.38	4.76	5%	25%	84%	99%	
**MARB**	rs109878735	28	5837959	5.06	0.07	3.51	7.10	100%	100%	100%	100%	
**MARB**	rs135729785	2	5635260	4.22	0.05	3.07	5.32	98%	99%	100%	100%	
**MARB**	rs42965385	2	16581032	4.38	0.06	3.13	5.53	96%	100%	100%	100%	
**MARB**	rs41639690	1	20312624	4.28	0.06	3.04	5.56	95%	99%	100%	100%	
**MARB**	rs41926443	19	56083804	3.97	0.05	3.08	5.46	93%	99%	100%	100%	
**MARB**	rs211134916	21	60548375	4.23	0.07	2.17	6.36	86%	94%	97%	99%	
**MARB**	rs3423095175	12	21560480	3.91	0.06	2.77	5.39	85%	95%	100%	100%	
**MARB**	rs42267605	26	49205001	3.99	0.06	2.54	5.93	82%	94%	99%	100%	
**MARB**	rs108986373	5	21979725	3.81	0.05	2.38	5.03	82%	95%	98%	100%	
**MARB**	rs109897238	27	817350	3.77	0.06	2.73	5.96	79%	91%	99%	100%	

Columns show the rs number (SNP id), chromosome (BTA), base pair position (BP), median –log10(*P*), standard error (SE), minimum and maximum –log10(*P*) across CV runs, and the percentage of runs in which each SNP was included in the top 50 to 1000 SNPs.

In contrast, the MARB GWAS (Fig. 3B) revealed a more dispersed pattern of association, with top SNPs distributed across multiple chromosomes rather than concentrated in a single region as observed for WBSF. The highest-ranking SNP for marbling (rs109878735, chromosome 28) had a median –log_10_(*P*) of 5.06 and was included in the top 50 in 100% of replicates. Other top SNPs were located on chromosomes 1, 2, 5, 12, 19, 21, 26, and 27, with slightly more variation in inclusion frequency across subsets, consistent with a polygenic trait architecture.

These results suggest that while WBSF prediction may be driven by a relatively small number of consistently high-ranking SNPs (particularly on chromosome 29), accurate prediction of MARB requires a broader sampling of markers, reflecting the larger number of small-effect loci contributing to variation in this trait.

### External validation of prediction models

**Fig. 4.**
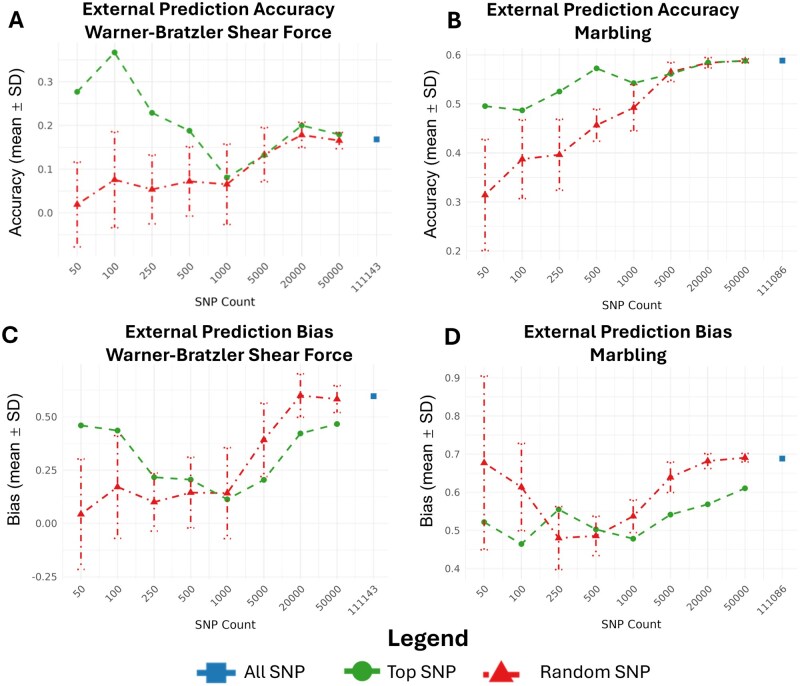
Accuracy and bias of genomic predictions across SNP subsets in external validation population. Prediction accuracy (panel A: WBSF, panel B: MARB) and regression slope (panel C: WBSF, panel D: MARB) across SNP subset sizes. Blue dots represent the full SNP panel, while lines show the results for the top-ranked SNPs (green) and the mean± SE for the random SNPs subsets (red) .

Figure 4 presents prediction accuracies from the external validation set, where GEBVs were estimated for the UF MAB cattle using the cross-validation population as a reference. As in the cross-validation analysis, both WBSF and MARB showed contrasting trends depending on SNP subset size and ranking strategy.

For WBSF (Fig. 4A), the highest accuracy was achieved with the top 100 SNP subset (0.36), which substantially outperformed both random subsets of the same size and the full SNP model (0.18 using all 111,143 markers). Even the top 50 subset outperformed the full model, reinforcing the cross-validation finding that a small set of highly informative SNPs can capture most of the predictive power for this trait. Randomly selected subsets of equivalent size yielded substantially lower accuracy, particularly below 5000 SNPs.

In contrast, MARB (Fig. 4B) exhibited a more incremental improvement in prediction accuracy with increasing SNP count. The full SNP model reached an accuracy of 0.60, but this was nearly matched by the top 50,000 subset (0.59) and top 20,000 (0.59) subset, suggesting diminishing returns beyond these marker densities. Notably, the top 1000 SNP subset al.eady outperformed the full set of 111,086 markers. Unlike WBSF, MARB did not show an advantage for extremely small subsets; instead, accuracy steadily improved with marker count in both top-ranked and random SNP sets, consistent with a polygenic trait architecture. Together, these external validation results corroborate the internal cross-validation trends: WBSF is largely explained by a few major loci, while MARB prediction improves with broader genome-wide information. Moreover, they demonstrate that strategic SNP preselection can yield equal or superior performance relative to genome-wide models, even when applied in independent populations.

When examining prediction bias in the external validation population, we observed that regression slopes for the smallest SNP subsets were similar to those from the cross-validation analysis for WBSF. However, unlike in cross-validation where bias steadily improved toward 1.0 with increasing SNP density, slopes in the external validation analysis remained well below 1 across all SNP subsets and never exceeded 0.5. These results indicate a stronger tendency for the model to overestimate GEBV differences when applied outside the Brangus CV population. In other words, although the model can rank UF MAB animals effectively, it systematically exaggerates the magnitude of their predicted genetic differences, even when large SNP panels are used.

### Marker-assisted prediction for WBSF

Given the strong predictive performance observed for WBSF using low-density SNP panels, we further evaluated a marker-assisted prediction approach using the single most significant SNP identified within each CV training population (Fig. S3).

In the CV population, the marker-assisted model achieved a mean prediction accuracy of 0.23 (Fig. S3A). This performance was lower than that obtained using the top 50 SNP subset (accuracy = 0.301). In contrast, prediction accuracy in the external validation population was effectively zero (–0.005), indicating limited transferability of single-marker models across populations (Fig. S3A).

Bias estimates further highlighted these differences. In cross-validation, the marker-assisted model exhibited a regression slope of 0.647, indicating better bias relative to the top 50 SNP subset (Fig. S3B). However, in the external population the regression slope approached zero (0.0009), consistent with the lack of predictive signal observed for accuracy (Fig. S3B). Together, these results indicate that while a single major locus can partially capture genetic variation for WBSF within the CV population, reliance on a single marker is insufficient for robust prediction across populations.

## Discussion

This study evaluated the effect of SNP preselection on genomic prediction accuracy using both cross-validation and external validation frameworks. The results demonstrate that even at low SNP panels competitive accuracies for traits can be achieved, however the genetic architecture of the trait plays a large role in the number of SNP needed.

For both traits analyzed, prediction accuracy generally increased with SNP set size, particularly for randomly selected subsets. This is consistent with previous studies using cross-validation frameworks, which report steady gains in accuracy as more SNPs are included, likely due to a greater genomic coverage and improved tagging of causal variants (Meuwissen et al. 2001; Hay et al. 2018). Our findings align with this trend, especially for random SNP subsets where no attempt is made to enrich for causal variants, thereby approximating the general case of denser genotyping arrays or imputed datasets (van Binsbergen et al. 2015). However, it is important to note that this relationship is not always linear; multiple studies have found that very high-density panels (eg 770K) or whole-genome sequence data often perform similarly or worse than standard 50K arrays, likely due to the inclusion of many non-informative or population-specific variants that introduce noise (Harris et al. 2011; van Binsbergen et al. 2015; Wu et al. 2016).

SNP preselection based on association results produced very different outcomes for the two traits. For WBSF, strong prediction accuracy emerged even when using small SNP panels. This outcome was driven by a large-effect QTL on BTA29 that includes *Calpain 1 (CAPN1)*, a gene encoding a calcium-dependent cysteine protease central to postmortem muscle proteolysis and meat tenderization. (Page et al. 2002; White et al. 2005; Mateescu et al. 2017). The strong signal on BTA29 observed in this study is consistent with decades of research identifying *CAPN1* as a major determinant of beef tenderness (Page et al. 2002; White et al. 2005; Schenkel et al. 2006; McClure et al. 2012; Sun et al. 2018). Rather than representing a novel discovery, our results quantify how this known genetic architecture translates into genomic prediction performance when marker density is aggressively reduced. This QTL was consistently selected in every replicate, providing strong internal and external validation. One unexpected result was the lack of a significant signal near *Calpastatin (CAST)* on BTA7. This QTL is widely documented as a major contributor to tenderness and was included in early MAS panels such as GeneSTAR (Schenkel et al. 2006; Johnston and Graser 2010; McClure et al. 2012; Leal-Gutiérrez et al. 2018a). Closer inspection of the ARS-USMARC-116 marker previously associated to WBSF in the MAB population revealed a clear population-specific pattern (Leal-Gutiérrez et al. 2018a). Although allele frequencies of this marker were comparable between the Brangus (MAF = 0.16) and MAB (MAF = 0.19) populations, the marker was not significant in Brangus (*P* > 0.122) but showed strong significance in the MAB population (*P* < 0.001). Furthermore, no additional SNPs in the *CAST* region reached significance in Brangus. Together, these results indicate that the underlying QTL either does not segregate in the Brangus population or that no SNP in the panel is in LD with the causal mutation in the Brangus population. In line with this, previous reports have found that *CAST* was not significantly associated with WBSF (Curi et al. 2010; Sun et al. 2018). Although *CAST* and *CAPN1* are considered large-effect loci, WBSF remains a polygenic trait. In crossbred cattle, *CAST* and *CAPN1* have been reported to account for 3.97% and 5.41% of the genetic variance in WBSF, respectively (Fonseca et al. 2022). For genomic prediction, the presence of a major QTL on BTA29 enabled accurate WBSF prediction using panels of only 50 to 100 selected SNPs. Beyond this range, prediction accuracy declined as additional SNPs were added, reaching a minimum before recovering once panel size approached roughly 5000 SNPs, where accuracy began to improve again (Figs. 2A and 4A). This intermediate decline likely reflects redundancy among top-associated SNPs, many of which tag the same QTL and contribute to overfitting. The LD structure surrounding the BTA29 signal (Fig. S2) supports this interpretation, as the most significant SNPs clustered within 42.4–43.4 Mb and formed a strong LD block (r^2^), indicating that many selected markers are effectively redundant. In contrast, MARB displayed a genetic architecture typical of complex polygenic traits, influenced by many small-effect loci distributed across multiple chromosomes as can be seen in Fig. 3B. This pattern aligns with prior GWAS in AngusxBrahman cattle crosses (Zayas et al. 2024; Baneh et al. 2025), underscoring the importance of broad genomic coverage for accurate prediction. Because the causal effects are small and dispersed, larger marker panels provided better tagging of segregating variation. Accuracy did not begin to plateau until the panel included roughly 20,000 SNPs. Across both traits, selected SNP panels outperformed random panels at low to moderate densities because they captured trait-relevant signal more efficiently. However, once SNP counts exceeded about 20,000, accuracy for selected and random panels converged for both MARB and WBSF. At this point, marker density was high enough that even random subsets captured broad genomic relationships and a substantial portion of the underlying polygenic variance, leading to similar prediction performance.

For WBSF, the influence of the BTA29 *CAPN1* locus shaped the entire bias profile. At very small SNP densities, selected panels had better bias values compared to random panels because they consistently included the large-effect QTL and aligned GEBVs more closely with the phenotype scale. As density increased from about 100 to 500 SNPs, bias worsened among the selected panels. Most of the added SNPs were in high LD with the same underlying *CAPN1* signal, which inflated the QTL’s effect estimate rather than adding independent information. In contrast, random panels improved steadily with increasing density. Once random sets reached about 1000 SNPs, they began to outperform selected sets in terms of bias because they captured more polygenic background and better approximated the genomic relationship structure of the population.

MARB showed a different pattern because it lacks a dominant QTL. Both selected and random panels began with similar bias at low SNP densities. As density increased, selected and random panels improved almost linearly because each additional SNP added small, independent contributions that helped reconstruct the underlying genetic variance. Selected panels improved more slowly and eventually lagged behind the random sets. The selected subsets tended to repeatedly include the same small-effect loci across replicates, which led to mild but persistent inflation of GEBVs.

While trends for WBSF and MARB were consistent between both CV and external populations, there are some key differences regarding the magnitude of accuracy and bias. Our result showed that MARB and WBSF accuracies were higher and bias (overestimation of GEBV; slope <1) was more severe in the external UF MAB population than in the Brangus CV set. For MARB, accuracy in the UF MAB population reached 0.60 compared with 0.382 in Brangus, and the best bias estimates using the full SNP panel were 0.843 in Brangus and 0.695 in UF MAB. WBSF showed the same pattern: the highest CV accuracy in Brangus was 0.303, whereas accuracy in UF MAB reached 0.36, yet bias dropped from 0.959 in Brangus to 0.621 in UF MAB, indicating pronounced overestimation of the GEBV in the UF MAB.

Across-population prediction theory may explain these shifts. When SNP effects estimated in one population are applied to another with different allele frequencies and LD structures, changes in both accuracy and dispersion are expected (Wientjes et al. 2015). The observation that loci like *CAST* were significant in MAB but not in Brangus suggests that the two populations differ in their local LD patterns and segregating QTLs. Most prior work has examined purebred reference populations predicting crossbreds; to our knowledge, there are no studies that estimate SNP effects in a composite breed like Brangus to predict animals spanning a wide spectrum of Angus and Brahman ancestry. Some studies have shown that including crossbred alongside purebreds generally results in gains in accuracy but inconsistent improvements in bias for crossbred predictions (van den Berg et al. 2019; Khansefid et al. 2020; Misztal et al. 2022). Bias is more reliably improved when models incorporate breed-specific allele frequencies or breed-of-origin information (Guillenea et al. 2023; Warburton et al. 2023).

Our previous work showed that the Brangus population used here shares relatively uniform admixture proportions (about 60 to 80% Angus) and is genetically related to the UF MAB herd (Zayas and Mateescu 2025). This Brangus population does represent a much narrower segment of the Angus–Brahman spectrum, thus SNP effects estimated within the population are going to capture modest within-composite breed contrasts and only slight ancestry-related differences. When these effects are applied to the UF MAB population, which spans a much broader ancestry gradient, the same contrasts seem to become amplified. Effect estimates generated under the more restricted ancestry structure of Brangus are projected onto animals with more extreme ancestry combinations, which widens the predicted genetic differences.

Given the well-documented breed differences in MARB and WBSF between Angus, Brahman, and their crosses, these amplified contrasts help the model rank animals in the UF MAB population more effectively (Elzo et al. 2012). At the same time, this difference may be exaggerated in our model, because the effects were estimated in a more homogeneous population with smaller underlying contrasts. In summary, we believe that the higher accuracies for MARB and WBSF in the UF MAB population reflect improved ranking across a wider genetic spectrum, while the lower regression slopes reflect inflated GEBV estimation.

A different methodological difference between analyses is the phenotype adjustment strategy: phenotypes in the Brangus cross-validation were adjusted for contemporary group, whereas phenotypes in the external population were adjusted for year. Differences in fixed-effect structure can alter the residual variance and, in turn, affect estimates of accuracy and bias in the external population.

Together, these results of accuracy and bias for the CV and external population highlight both the opportunities and challenges of applying genomic prediction models across more homogenous and heterogeneous populations. They reinforce the importance of genetic relatedness between the training and target populations, a point long established in genomic prediction literature (Habier et al. 2007; Clark et al. 2012). Notably, we found that in the external UF MAB population, smaller subsets of significant SNPs sometimes improved accuracy for WBSF and performed comparably to larger or full panels for MARB, although these gains also came with stronger bias.

Although superficially similar to MAS, our approach differs because it does not rely on fitting a small number of fixed SNP effects. Instead, it incorporates many statistically supported SNPs as random effects within a GBLUP/SNPBLUP framework. This regional tagging strategy can provide greater robustness across populations where causal variants are unknown, weakly captured, or inconsistently linked. This distinction becomes especially important when examining the MAS results for WBSF. Within the Brangus CV population, the top single marker (across different folds) produced a reasonable accuracy of about 0.230 (Fig. S3). However, the same markers performed poorly in the UF MAB population, with a mean accuracy of −0.005 (Fig. S3). In other words, although the top SNP was informative within the Brangus population, its predictive value did not transfer to the external population. Including a broader set of statistically supported SNPs mitigates this issue because the model captures regional LD patterns rather than depending on a single marker whose LD phase may not hold across populations.

GWAS-informed SNP prioritization is conceptually related to weighted GBLUP approaches, which assign differential importance to markers based on prior information or association strength (Wang et al. 2012). While weighted GBLUP modifies the genomic relationship matrix through marker weighting, SNP prioritization/selection methods focus on marker selection while retaining equal weighting among the included SNPs. These strategies are similar but can be complementary. Future work could integrate GWAS-based SNP prioritization with weighted relationship matrices to potentially further improve prediction accuracy and robustness.

Our results demonstrated that well validated and biologically informative SNP subsets provided strong ranking ability for key traits even when full genomic information was not used. For WBSF, a highly reduced panel of 50 to 100 SNPs, representing approximately 0.045% to 0.09% of the full 111,143-SNP panel, achieved prediction accuracies of about 0.301 in the Brangus CV population and 0.36 in the external population, matching or exceeding the accuracy of the full SNP panel (0.303 in CV and 0.18 in the external population). In contrast, marbling showed a stronger dependence on marker density. Prediction accuracy increased from 0.33 with 500 SNPs to 0.38 with the full panel in the Brangus CV population, and from approximately 0.50 with 50 SNPs to approximately 0.59 with the full panel in the external population. Despite this trend, subsets of 50 to 1000 SNPs, representing 0.045% to 0.90% of the full panel, still achieved competitive prediction performance relative to the full array. Comparing these results to those of the MAS also showed that including multiple of the top SNPs for large effect QTLs like the *CAPN1*, showed much better accuracy. Largely due to the differences in LD and allele frequencies between populations.

An important limitation of this study is that all evaluations were conducted using single-trait prediction models. Selection decisions based on a single trait can result in unfavorable correlated responses in other economically important traits. In practice, genomic predictions are applied within multi-trait selection indices that balance meat quality, growth, fertility, and adaptability. As such, the utility of SNP preselection should ultimately be assessed in a multi-trait context, where genetic correlations among traits are explicitly accounted for. Future research should evaluate the performance of SNP-preselected models within multi-trait genomic frameworks, including their application to higher-density genotyping platforms, and directly compare these approaches with standard weighted GBLUP-based models.

## Conclusion

These results demonstrate the efficiency with which informative genomic regions can capture prediction accuracy for traits influenced by major loci. GWAS-informed SNP prioritization provides a framework for understanding marker density requirements, guiding marker weighting strategies, and designing research-oriented or targeted genomic tools. While not intended as a replacement for high-density commercial platforms, this approach highlights opportunities to optimize genomic prediction pipelines for specific traits and populations.

## Supplementary Material

txaf175_Supplementary_Data

## Abbreviations:

BTA: Bos taurus autosome
*CAPN1*: calpain 1 gene
CG: contemporary group
CV: cross-validation
G×E: genotype × environment interaction
GBLUP: genomic best linear unbiased prediction
GEBV: genomic estimated breeding value
GWAS: genome-wide association study
LD: linkage disequilibrium
MAB: multibreed Angus–Brahman (UF resource herd)
MARB: marbling score
MAS: marker-assisted selection
QC: quality control
QTL: quantitative trait locus/loci
SNP: single nucleotide polymorphism
UF: University of Florida
USDA FSIS: United States Department of Agriculture Food Safety and Inspection Service
WBSF: Warner–Bratzler shear force
