# Supplementary material for: Genomic prediction of beef quality using GWAS-prioritized markers
Source: Transl Anim Sci. 2026 Jan 6;10:txaf175. doi: 10.1093/tas/txaf175 (PMC12861978; doi:10.1093/tas/txaf175)
Supplement: txaf175_Supplementary_Data [file txaf175_supplementary_data.docx]

**Supplementary Figures**

**
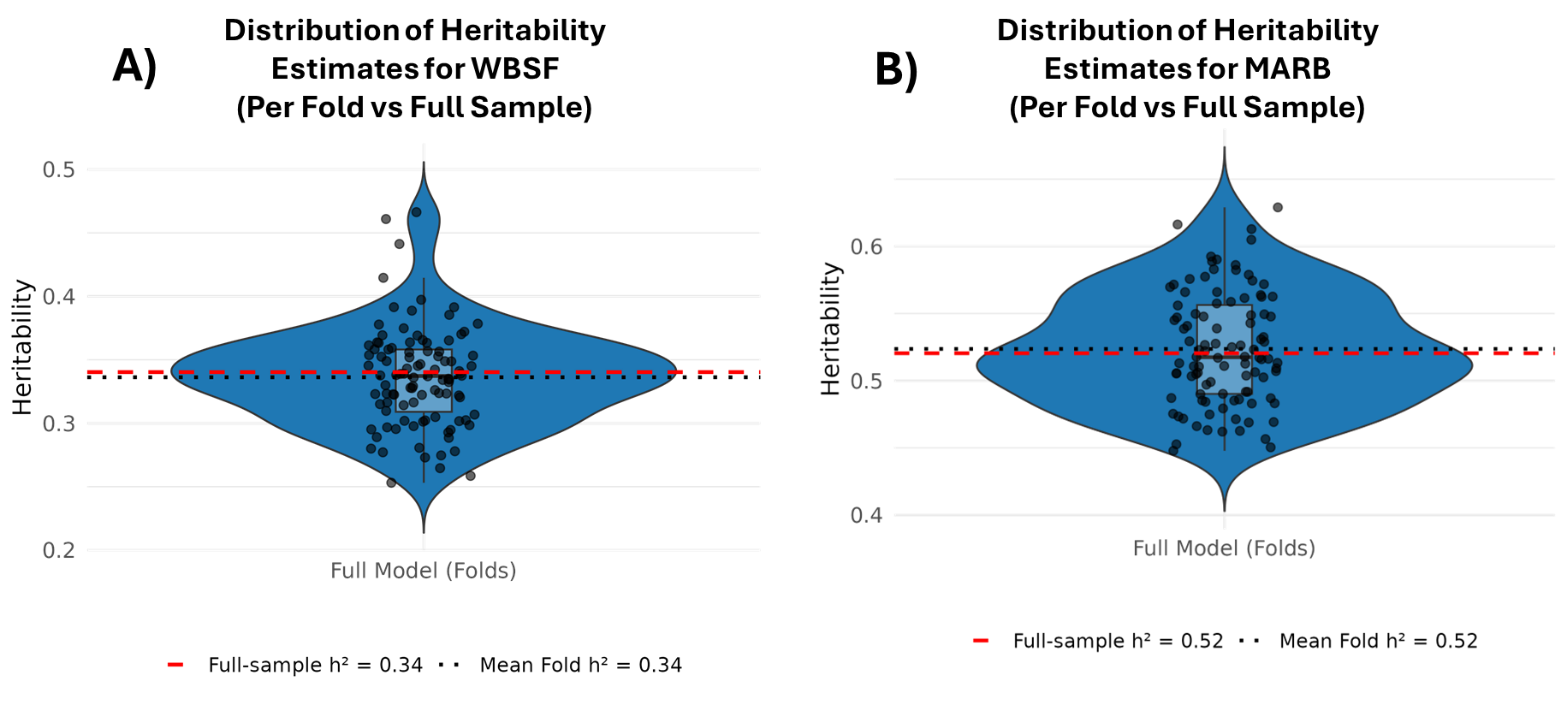
**

Supplementary Figure 1) Violin plots of heritability estimates across all folds and replicates in the cross-validation scheme for A) Warner–Bratzler shear force and B) marbling. The red dotted line indicates the full-sample heritability estimate, and the black line shows the mean estimate across folds and replicates.

**
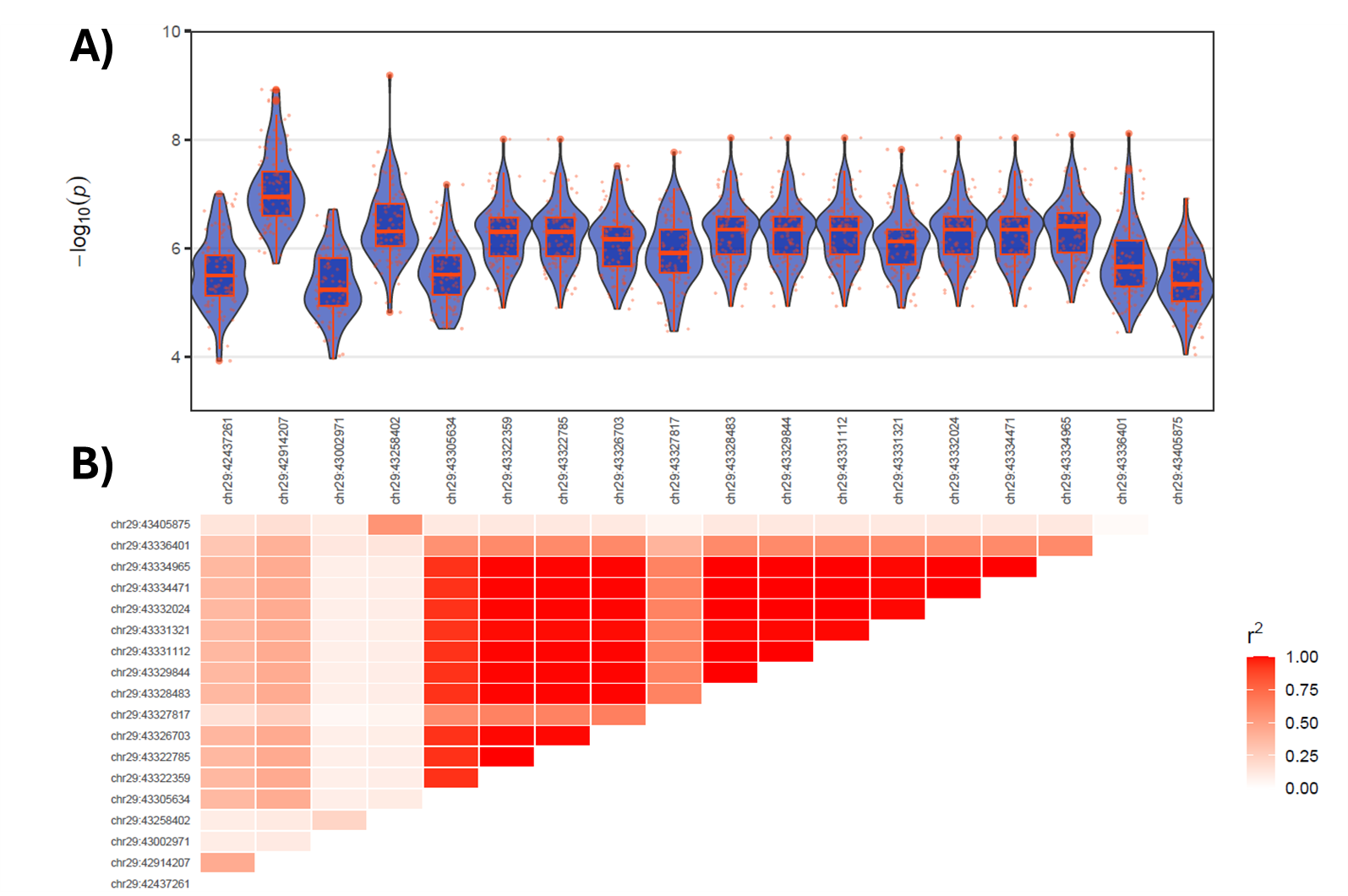
**

Supplementary Figure 2) A) GWAS results for significant SNPs on BTA29:42.4–43.4 Mb, shown as violin plots where each SNP is represented by a violin and each dot corresponds to the –log₁₀(p-value) from a given fold and replicate. B) Linkage disequilibrium among these SNPs, shown as pairwise r² values.


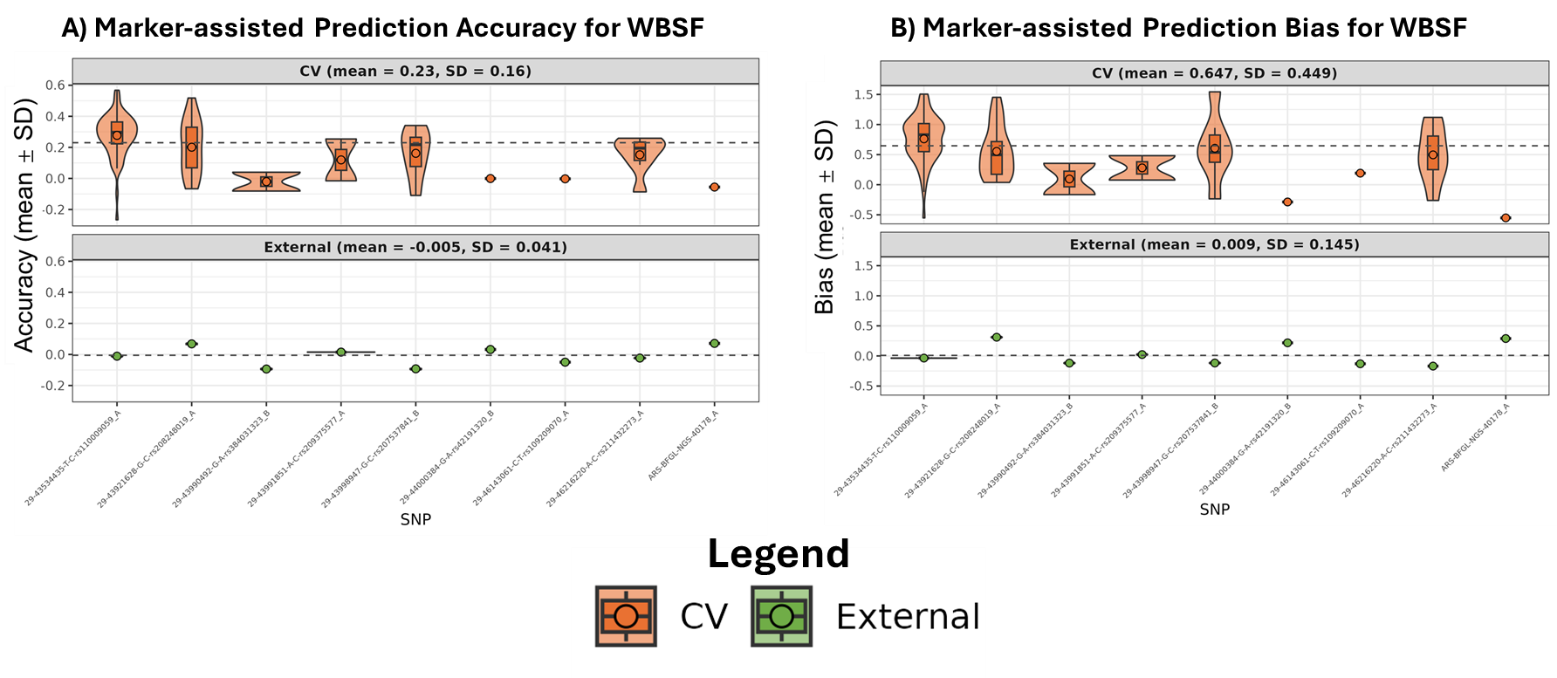


Supplementary Figure 3) A) Accuracy measures of SNP used for Marker-assisted prediction in CV population and external populations. B) Bias measures of SNP used for Marker-assisted prediction in CV population and external populations.
